# Poised for a dividend? Changes in the life trajectories of India’s young women over the past two decades

**DOI:** 10.1371/journal.pone.0242876

**Published:** 2020-12-28

**Authors:** Shareen Joshi, Kakoli Borkotoky, Abhishek Gautam, Nitin Datta, Pranita Achyut, Priya Nanda, Ravi Verma

**Affiliations:** 1 Associate Professor of International Development, Edmund Walsh School of Foreign Service, Georgetown University, Washington, DC, United States of America; 2 Technical Specialist, International Center for Research on Women, Asia Regional Office, New Delhi, India; 3 Senior Specialist–Research and Programs, International Center for Research on Women, Asia Regional Office, New Delhi, India; 4 Senior Specialist–Monitoring and Evaluation, International Center for Research on Women, Asia Regional Office, New Delhi, India; 5 Director- Research and Programs, International Center for Research on Women, Asia Regional Office, New Delhi, India; 6 Senior Program Officer, Bill & Melinda Gates Foundation, New Delhi, India; 7 Regional Director, International Center for Research on Women, Asia Regional Office, New Delhi, India; University of Botswana, BOTSWANA

## Abstract

This paper examines recent changes in the life trajectories of Indian women. We use data from four major national population surveys that span the years 1998–2016. We look at several cohorts of women across the states and regions. We compare decisions related to education, marriage, childbearing and participation in the labor force. Though there is considerable diversity across states and regions, as well as religious groups, we find some consistent patterns that emerge everywhere. First, educational attainment and the age at marriage have been steadily increasing. Women who do not complete secondary school are more likely to marry early. Second, caste and religion (rather than education) play a significant role in decisions after marriage, such as the timing of births, the use of contraception and labor force participation. Third, women from disadvantaged communities continue to have very different life trajectories than other social groups. They are more likely to use contraception and participate in the labor force. Lower levels of schooling also appear to exacerbate the disadvantages of social identity. The pace of these changes varies sharply across states as well as regions of the country.

## Introduction

India is banking its future on its “demographic dividend”–a temporary surge in the ratio of the working-age population to the dependent population caused by a sustained decline in fertility and improvement in child survival, that can potentially increase labor supply, GDP, savings and investment [1–4]. The realization of this dividend however, depends heavily on decisions made by young women and the policies designed to support them. Greater opportunities for education and employment for example, create favorable conditions for women to delay marriage, reduce fertility, reallocate labor away from unpaid work towards market activities, make greater investments in the quality, rather than the quantity of their children and participate in public life [5, 6].

A large literature demonstrates that Indian women face complex familial, social, economic and political barriers that limit their opportunities [7–9]. Only 60 percent of adult Indian women aged 15 and above are literate–this is well-below the world average of 82 percent [10, 11]. Child marriage remains common–though the numbers have been declining in recent years, over a quarter of Indian women still marry before the legal age of 18 [12]. Women contribute only 18 percent of Indian GDP and account for only 25 percent of the formal labor force [13]. It is also striking that even though policy makers have been expanding women’s opportunities and educational attainment has been improving, India’s female labor force participation rate has been falling [14–18].

The empirical literature on gender inequality in India also highlights the importance of geographic diversity: variations in outcomes such as education, marriage and fertility that are seen across India’s regions, states, districts and towns are often as significant as between the developed and the developing world and also remarkably persistent over time [19–27]. There are historical differences across these regions, but states have also showed significant variations in the policies that expand opportunities for women [8, 28].

Most of the recent existing empirical literature on spatial variations in gender inequality in India uses data from national surveys such as the National Family Health Surveys (NFHS), District Level Household Surveys (DLHS) and Indian Human Development Survey (IHDS), which are all nationally representative population surveys that permit sub-national analysis. With the exception of the latest round of the NFHS however, these surveys were conducted more than a decade ago, and thus provide no insights into the life decisions of India’s youngest women–the cohort on whom India’s demographic dividend now hinges. This is a critical gap. In this paper we use four major national population surveys that span the years 1998–2016 to construct a broad descriptive overview of the key decisions in the lives of young women. We also use data from the third and fourth wave of the National Family Health Survey, collected in 2005–06 and then 2015–16 to explore the deeper drivers of marriage, fertility and labor market participation in a statistical framework.

Our approach is based on a life-trajectory model. We compare younger cohorts (age 15–24) to older cohorts (ages older than 25). Several important findings emerge from this analysis. First, we find that there has been a slow increase in both educational attainment and the age at marriage all across India. According to the most recent data, more than half of Indian women are now married *after* the age of 18. Moreover, young women aged 15–24 are more likely than ever before to complete secondary schooling. There are however, dramatic variations across states. The gap between the best- and worst-performing states has remained relatively constant over time. In regressions of microdata we find the persistence of a strong positive correlation between completion of secondary school and delayed marriage (to past 18), but a weakening of the impact of caste and religion on the probability of being married before 18. This increase in the age at marriage and schooling attainment, that is largely decoupled from the role of caste and religion is a noteworthy development for modern India.

A second finding that emerges from our analysis relates to fertility. We see that though women are delaying marriage, they seem to have children very quickly after their union. The age at first birth has remained almost constant throughout the rounds of the survey. Moreover, the first birth interval–the number of months between marriage and birth of a first child–has been declining almost everywhere. This may also contribute to the well-known decline in the usage of modern contraception in some parts of India, though it may also be driven by other factors such as changes in the age at the consummation of marriage and migration from a woman’s natal to her husband’s home. Delays in marriage and completion of education seems to increase the pressure on a woman to bear children quickly after marriage. This may also account for the decline in female employment in the most recent round of the survey. The contrast between our results on the age at marriage and education on the one hand and the timing of births on the other is noteworthy, for it suggests that “dividend” of education and delayed marriage is not necessarily being seen during motherhood.

Finally, we demonstrate that even though women in the 15–24 age-group have greater levels of educational attainment than the generations before, they are not more likely to be employed. We conjecture that delays in marriage, lower utilization of modern contraception and shorter first-birth intervals together place constraints on female labor-force participation. Given that India already has one of the lowest levels of female labor-force participation in the world and seeking to benefit from its demographic dividend in the next 50 years, this is noteworthy.

In the next three sections we describe our data, conceptual framework and methods. In the subsequent section we present our findings and discuss the implications of these findings. The final section concludes.

## Conceptual framework

Our overall approach is based on life-course theory [29–31]. This interdisciplinary approach is widely used, either directly or indirectly, in public health, demography, sociology, public policy, economics and history. It emphasizes the structural, social and cultural contexts in which decisions are made, and the continued impact of those decisions long after they are made. In studies of health for example, the life-course approach is used to study the impacts of critical events in gestation, childhood, adolescence, young adulthood and midlife on the incidence of chronic diseases throughout life.

Ideally, life-course analysis requires panel data on a number of different cohorts–the decisions made early on in life can then be directly linked to later outcomes–comparisons across cohort provide evidence of change. In this study however, we are limited to pooled cross-sectional data that is collected for heterogenous groups of women in each survey. We must thus adapt this framework to study the shifts in major life events across cohorts of women. We emphasize several key events in a woman’s life trajectory: Completion of secondary school, age at marriage, the decision to use contraception, and formal employment.

Our focus on the completion of secondary school is motivated by the persistence of high dropout rates for girls at this stage of education. According to recent estimates, a quarter of all Indian girls drop out of school during the years of secondary school [11]. Numerous recent studies suggest that drop-out rates are largely driven by early marriage, adolescent pregnancy, the cognitive challenges induced by early-childhood malnutrition and stunting, family concerns about the distance to school or the high indirect costs of schooling [8, 32–34].

Next, we focus on the age at marriage. For many women in India, this decision is interlinked with the completion of secondary school [33, 35, 36]. In North India, there has been a strong preference for early marriage for much of its recent history [28]. Most communities practice patrilocal exogamy, which historically required women to migrate from their natal home to their husband’s home, leaving inheritance rights to their brothers [28, 37, 38]. This transition is associated with significant vulnerability for women. The shift results in in-laws gaining considerable power over a woman’s decisions related to employment, utilization of health care, and access to and use of contraception [9].

Once a woman is married, decisions about contraception, participation in the labor market and childbearing are all deeply interlinked and influenced by societal and familial factors. A large literature shows detrimental effects of early marriage on the health, education and employment opportunities of young women [33, 35, 36]. Women’s mobility and agency may both be constrained as a bride in a new home. Her access to health care and contraception for example, is likely to be driven by her in-laws preferences as well as the conditions in their geographic location. The availability of health care and labor market opportunities vary significantly throughout India. And even within a single location, attributes such as caste, class and social status can constrain women. Existing literature suggests that women from disadvantaged castes are less likely to complete school, marry early, work in the labor force and bear children at a young age [39, 40].

Though it is not explicit in the framework, the life-course approach used here emphasizes that women’s opportunities are also likely to be shaped by government policies. Education opportunities for adolescent girls vary throughout the country [8, 32]. For married women, family-planning programs vary in their availability, intensity as well as methods of targeting couples. In some states and even districts, we see services such as family planning are targeted towards women in specific communities [41–43]. Later in the paper, we address these variations through a fixed-effects statistical model.

We emphasize that the analysis in this paper will be largely descriptive. We do not seek to estimate the causal impacts of any specific policies or programs. Instead, we examine the temporal variations in key variables and use a multiple linear regression model to examine the determinants of the ages at marriage and birth, the participation in labor markets and the adoption of contraceptives. Our explanatory variables in these statistical models are age, education, caste, religion, parity (where relevant) and state/region. Our main objective is to gain insights into the broad changes in the lives of women at a critical juncture in India’s demographic transition.

## Data

This study draws on national population-based household surveys that have been used to measure national and sub-national health outcomes in India for the past 20 years. We combine data from four rounds of Demographic and Health Survey (DHS) which is known as National Family Health Survey (NFHS-1:1992–93, NFHS-2:1998–99, NFHS-3: 2005–06; and NFHS-4: 2015–16). All these surveys are (almost) nationally representative, cross-sectional surveys. The survey sizes are presented in Table 1. Given that the first three waves of the survey were representative at the state-level, while the fourth round was representative at the district-level and therefore larger than the previous rounds, we focus on state-level estimates to examine aggregate trends over time. We focus on the last two surveys in detail because they are more relevant to the present time and also cover the period during which demographic transition began.

**Table 1:** Sample sizes and coverage of the four surveys.

*Survey round*	*Years*	*Number of households*	*Number of women*	*Age-range of women*	*Marital status*
*NFHS 1*	1992–1993	88,562	89,777	13–49	Ever-married
*NFHS 2*	1998–1999	91,196	91,000	15–49	Ever-married
*NFHS 3*	2005–2006	109,041	124,385	15–49	All
*NFHS 4*	2015–2016	568,200	625,014	15–49	All

Aggregating surveys at the state-level presents several challenges. First, the four surveys use different sampling frames. Rounds 1 of the NFHS used the 1991 census, the NFHS 3 used the 2001 census and the NFHS-4 used the 2011 census. The creation of new states and districts over the study period led to significant changes in these sampling frames. For example, three new states were created in the year 2000: Chhattisgarh (carved out of Madhya Pradesh), Uttaranchal (carved out of Uttar Pradesh and renamed Uttarakhand in 2007) and Jharkhand (carved out of Bihar). Telangana was established in 2014 (carved out of Andhra Pradesh). Given that new states largely emerged in response to development challenges in poorest areas of large states such as Bihar, Madhya Pradesh and Uttar Pradesh, this change in sampling frame affects the representativeness of state-level samples. One strategy of addressing this is to rename these states to the old parent states, but even here, population-level averages are still unlikely to be a reliable measure to construct or interpret a trend because the data was gathered differently, with a different district-level sampling frame. To overcome this challenge, our analysis focusses on cross-sectional analysis rather than the construction or interpretation of trends across the surveys.

Second, there is the challenge of respondent selection. The surveys differed in inclusion criterion for respondents based on their age and marital status [44]. The first two surveys only gathered detailed data on ever-married women while the later surveys gathered information on all women in those age-groups. This study focused on currently married women aged 15 to 44 since this age group was common in all the surveys. In our study the proportion ‘never-married’ is calculated from household member information. We divide the sample into age-cohorts and compare these groups across a range of indicators. The groups that are of most interest to us throughout the paper are the youngest women in the sample: women aged 15–24. We compare these groups to women in older age-groups throughout the paper.

Finally, there is the issue of variability in the data quality over time. The four surveys vary in the number of respondents, the length of the questionnaire, the protocols about privacy, and the implementing partners. These issues are particularly significant in the fourth and most recent round [45]. This sample was five times bigger than the first round, and it featured triple the number of questions. The larger sample size and complexity of the survey resulted in many changes in the criteria of selection of field organizations and implementation strategies at the state-level. To minimize the challenges of comparing data across rounds, we rely as much as possible on the women’s questionnaire and the simplest questions that were consistently asked across rounds, in a variety of ways. We also refrain from pooling the samples together, relying on separate models for separate surveys. Most of our indicators were critical to the focus of these population surveys: age, marital status, educational attainment, the timing of births, employment status and the contraception. Among these indicators, issues of data quality are likely to be significant mainly for the questions about contraceptive usage–the changes in the protocols of privacy, enormous time-pressures on survey enumerators and the complexity of the protocols for this particular module may have resulted in many respondents coded as non-users [46].

Summary statistics of these variables are presented in detail for the survey rounds 3 (conducted in 2005) and 4 (conducted in 2015). Summary statistics of our key variables from survey rounds 3 and 4 are presented in Table 2.

**Table 2:** Summary statistics of key variables drawn from NFHS-3 and NFHS-4.

	*NFHS-3, 2005–06, Full Sample*					*NFHS-4, 2015–16, Full Sample*				
*Variable*	*N*	*Mean*	*Std*. *Dev*.	*Min*	*Max*	*N*	*Mean*	*Std*. *Dev*.	*Min*	*Max*
*Ever Married (1: Yes; 0: No)*	124,385	0.746	0.435	0	1	699,686	0.757	0.429	0	1
*Age Married*	93,774	17.573	4.220	1	45	527,536	18.206	4.287	1	49
*Age at Fist Birth*	83,118	19.747	3.867	10	45.083	433,531	20.382	3.927	10	49.417
*First Birth Below Age 18 (1: Yes; 0: No)*	83,118	0.362	0.481	0	1	433,531	0.273	0.445	0	1
*First Birth Interval (in month)*	83,129	25.311	23.496	0	353	437,750	28.334	25.846	0	481
*Employed (1: Yes; 0: No)*	124,385	0.351	0.477	0	1	122,351	0.234	0.423	0	1
*Use any form of contraception (1: Yes; 0: No)*	124,385	0.423	0.494	0	1	699,686	0.373	0.484	0	1
*Sterilized (1: Yes; 0: No)*	124,385	0.270	0.444	0	1	699,686	0.239	0.427	0	1
*Age*	124,385	29.160	9.494	15	49	699,686	29.831	9.763	15	49
*Age15-24 (1: Yes; 0: No)*	124,385	0.376	0.484	0	1	699,686	0.354	0.478	0	1
*Age18-24 (1: Yes; 0: No)*	124,385	0.262	0.440	0	1	699,686	0.247	0.431	0	1
*AgeOver25(1: Yes; 0: No)*	124,385	0.624	0.484	0	1	699,686	0.646	0.478	0	1
*Years of schooling*	124373	6.106	5.191	0	23	699,686	6.729	5.19	0	20
*Not Completed Secondary School (1: Yes; 0: No)*	124,385	0.166	0.372	0	1	699,686	0.202	0.401	0	1
*Muslim*	124,385	0.135	0.341	0	1	699,686	0.135	0.342	0	1
*SCST*	124,385	0.297	0.457	0	1	699,686	0.36	0.48	0	1
*Age 18–24 × SCST × NoSecSch*	124,385	0.070	0.255	0	1	699,686	0.066	0.248	0	1
*Age 18–24 × Muslim × NoSecSch*	124,385	0.033	0.179	0	1	699,686	0.028	0.165	0	1
*Age18-24 × SCST*	124,385	0.081	0.273	0	1	699,686	0.09	0.286	0	1
*Age18-24 × Muslim*	124,385	0.039	0.194	0	1	699,686	0.038	0.191	0	1
*Muslim × NoSecSch*	124,385	0.122	0.327	0	1	699,686	0.115	0.32	0	1
*SCST × NoSecSch*	124,385	0.270	0.444	0	1	699,686	0.309	0.462	0	1
*Parity (children ever born)*	124,385	2.064	2.046	0	16	699,686	1.88	1.818	0	17

Notes: Note that the variable “Age at marriage” is calculated based on only those female respondents who reported being married. “Not Completed Secondary School” is abbreviated to NoSecSch.

### State-level variations in key events

The first step of our analysis is to examine the trends in our key dependent variable at the state-level. We use sample weights provided along with the survey to construct state-level estimates of the key dependent variables for women in two age groups: 15–24 and 25+. The motivation behind combining two age-groups 15–19 and 20–24 into a single category is to reduce the impact of errors in self-reporting of life events in these two age-groups [47] This age-group is of greatest interest to us in the paper; first because these are the women whose adolescence has overlapped with the start of India’s demographic transition, and secondly, this is one of the least understood groups of women in India today. The paucity of health surveys in the past 12 years has meant that there is a serious gap in our understandings of their life decisions. The choice of the comparison group of women aged 25 or older is partly pragmatism, i.e. we wish to compare the 15–24 age-group to a single comparison group. But it is also based on the observation (which will be documented further in this section) that this group is almost entirely married and have entered the stage of motherhood. These categories enable us to contrast the outcomes for the youngest women with the average Indian woman of reproductive age in these population surveys. This contrast provides an initial view into the changes currently occurring in the youngest groups of the population.

**Fig 1:**
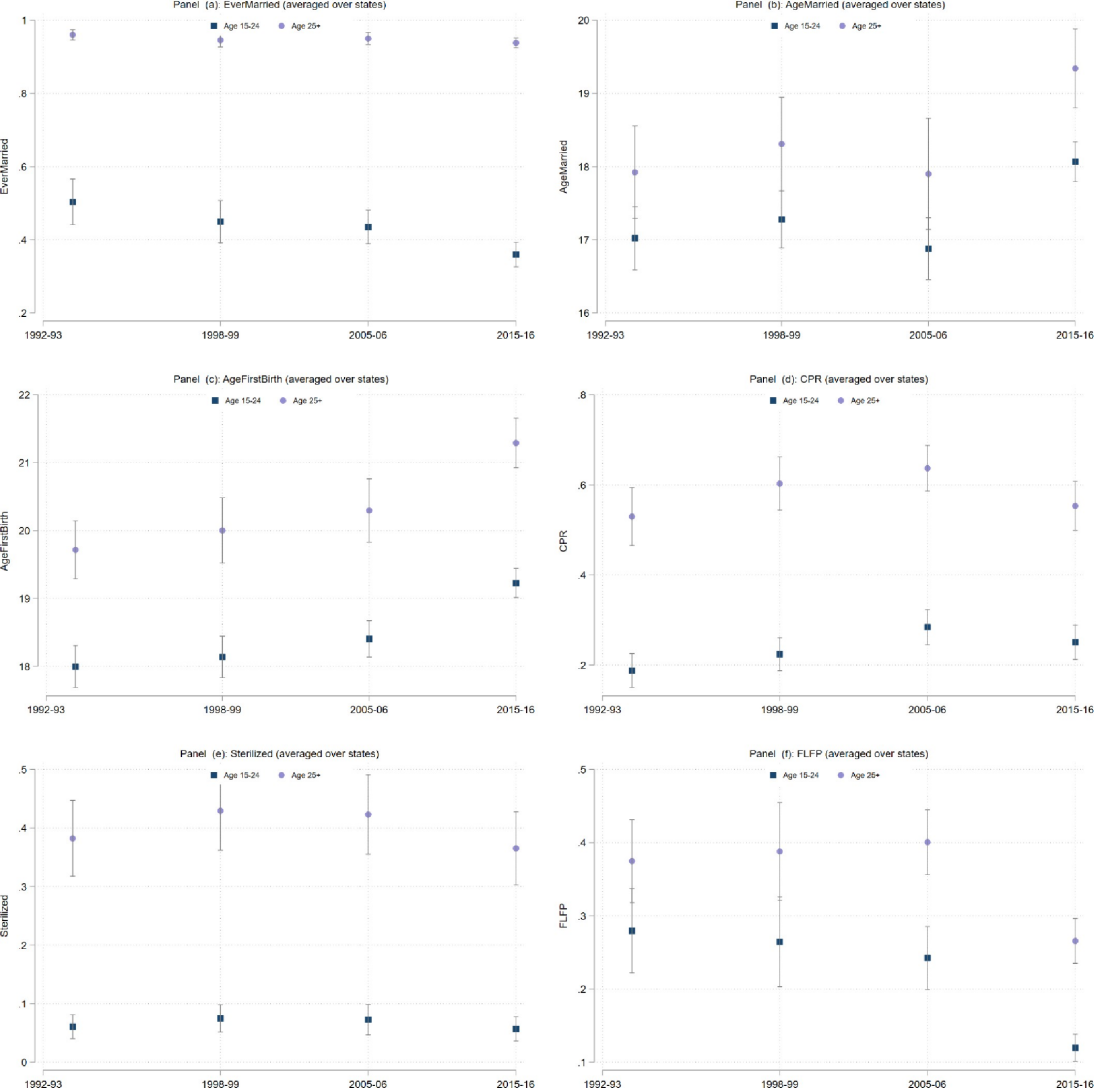
Average values and dispersion across states, by age-group and survey round.

**Fig 2:**
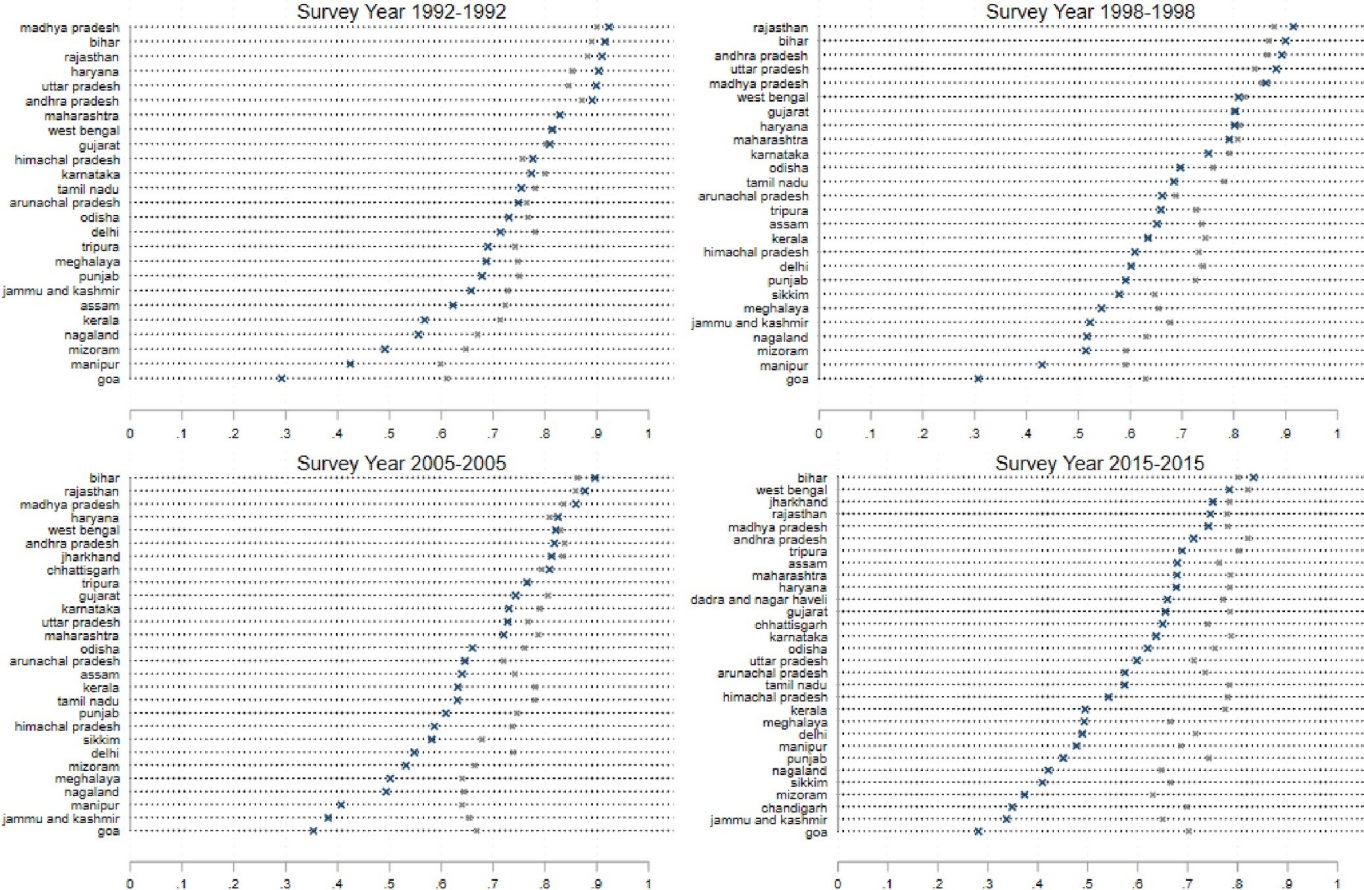
State level breakdown; percentage of women married, ages 18–24 (marked as ×) and age Age25+ (marked as *).

**Fig 3:**
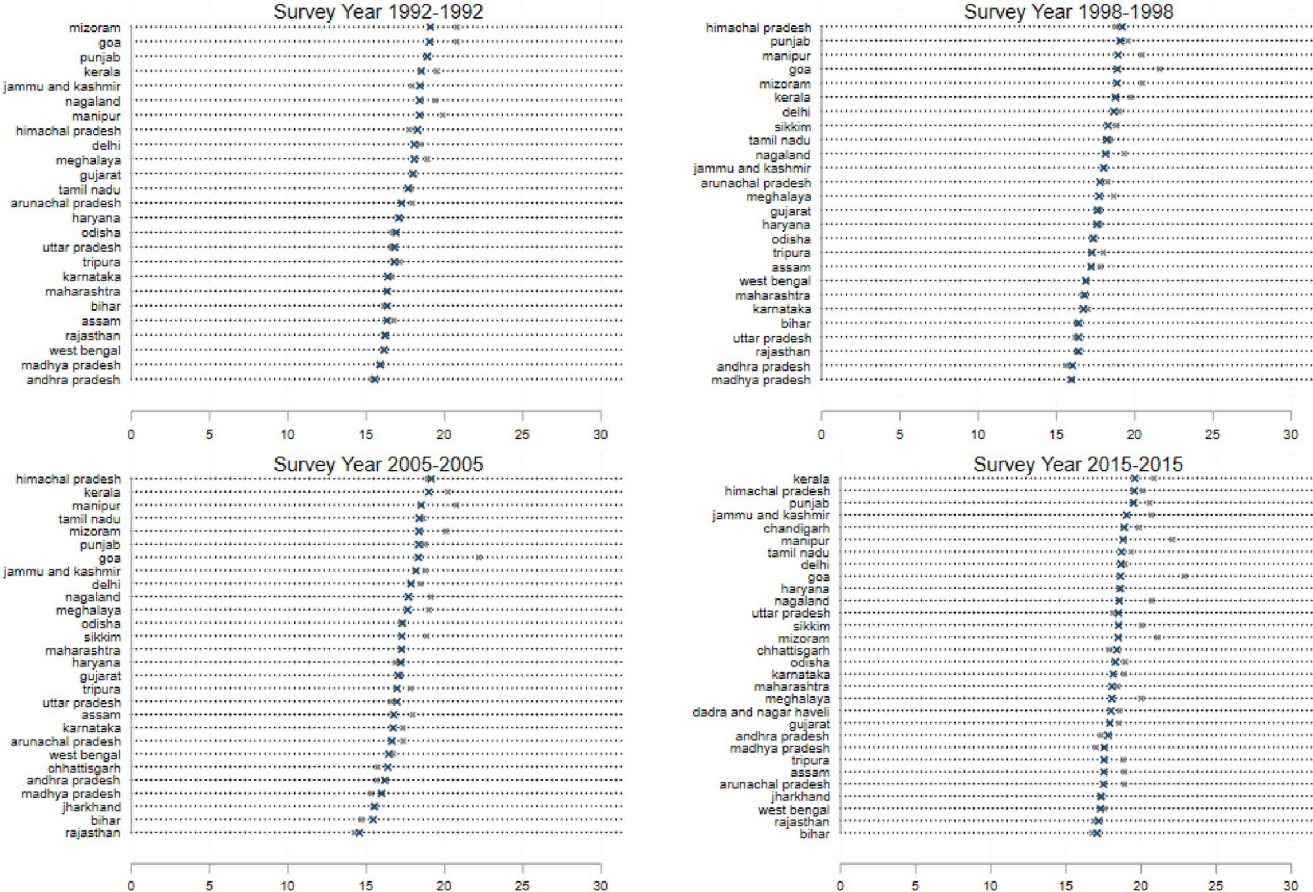
State level breakdown; age at marriage; percentage of women married, ages 18–24 (marked as ×) and age Age25+ (marked as *).

**Fig 4:**
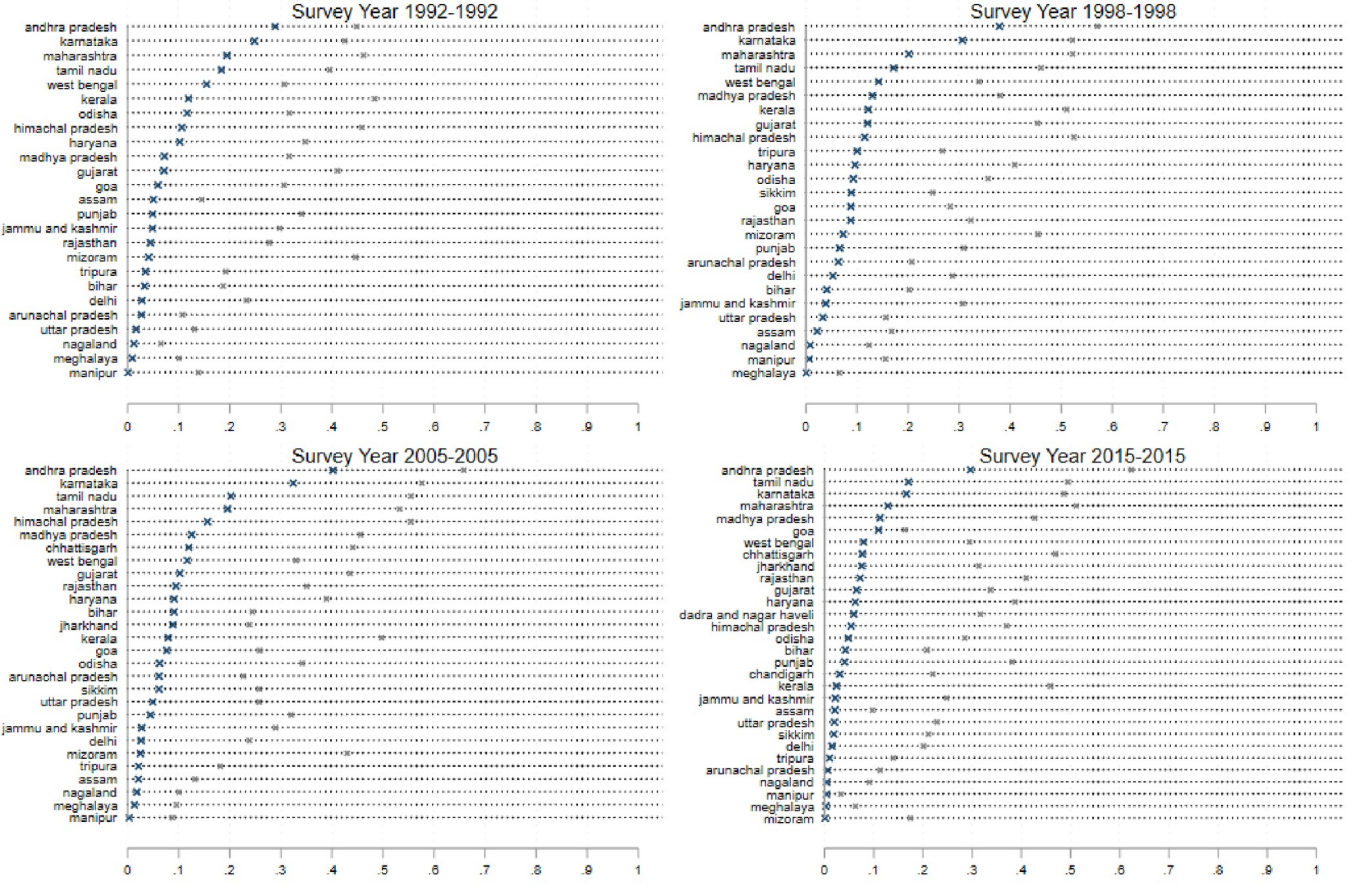
State level breakdown; sterilization; percentage of women married, ages 18–24 (marked as ×) and age Age25+ (marked as *).

**Fig 5:**
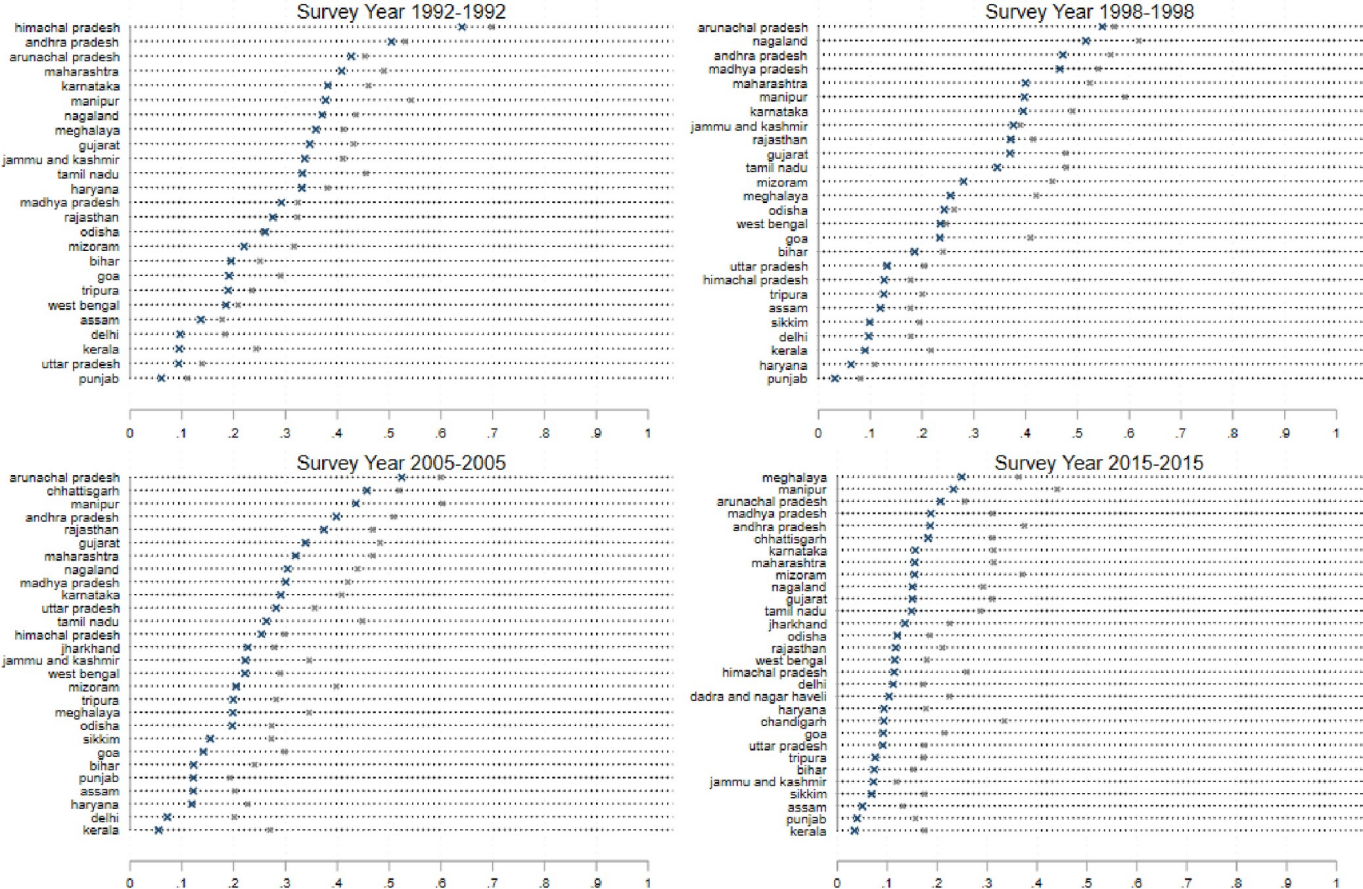
State level breakdown; employment; percentage of women married, ages 18–24 (marked as ×) and age Age25+ (marked as *).

The results are shown in Fig 1, which consists of several panels (a)—(f) depicting each of six dependent variables: (a) Ever Married: the fraction of women who report that they have ever been married; (b) Age Married: the average age at which a woman had her first marriage; (c) Age at First Birth: the average age at which a woman had her first birth; (d) the contraceptive prevalence rate (CPR): the fraction of women who report the reliance on any form of contraception; (e) Sterilization: the fraction of women who report being sterilized; (f) Female labor force participation (FLFP): the fraction of women who report that they were employed in paid work outside the household in the 6 months preceding the survey. Figs 2–5 present state-specific estimates for *some* of these dependent variables: Ever Married, Age Married, sterilization and employment. The CPR is omitted due to concerns over data, which we will explain below.

Fig 1, panel (a) suggests that marriage is almost universal in all the states of India throughout the sample period. In the average state, nearly 100% of women aged 25+ are currently married. Among the younger cohort however, the marriage rates are lower, and have been declining over successive rounds of the survey. In 1991, more than 50 percent of women aged 15–24 were married, but in 2015 this declined to less than 40 percent. In Fig 3, we see the actual estimates at the state level for all four rounds of the survey. Note that in 1991, with the exception of Goa and the states of the Northeast (Assam, Tripura, Nagaland, Mizoram, and Manipur), almost all the women aged 25 and older were married. There is much more variation across states in the younger age-group. In Bihar, Madhya Pradesh, Rajasthan, Haryana and Uttar Pradesh, we see almost four times more marriages in the 15–24 age group than in Goa, where only 20% of women in this age group are married. This persists in all four rounds of the survey, though the marriage rate for young women has fallen by 10% or more in these northern states between 1991–92 and 2015–16. This suggests that there has been an increase in the age at marriage in some states of India.

Fig 1, panel (b) presents state-level averages of the age at marriage. For these calculations, we restrict the sample of women to Ever_Married. In the sample of states, the age at marriage has increased over the past four rounds of the survey, but it was only in the most recent survey that the age at marriage crossed 18 for both the 15–24 age-group and the 25+ age-group. We also note that in all four survey rounds, the estimates for women aged 15–24 are well-below women aged 25+.

Fig 3 presents the estimates of age at marriage for individual states. The steep lines in this graph indicate the narrow range of this variable across the states of India–we must also emphasize that for the youngest women, we only measure this variable for women who were married at the time of the survey and thus do exclude women who do not marry in this age-group. We note that Himachal Pradesh in the North, Goa and Kerala in the South and most of the Northeastern states have consistently displayed the highest average ages at marriage in the sample. The northern states of Bihar, Rajasthan and Jharkhand have consistently been at the bottom of the ranking in all the survey rounds given the historical preferences for early marriage in the North [8, 28]. The gap between the states with the highest and lowest ages at marriage has remained remarkably stable in the first three rounds of the survey, but in the 2015 sample, there appears to be some convergence around the age of 18 for the youngest age-group in the sample. It is important to note however, that despite this progress, 12 states of India still display average ages at marriage below the age of 18 for the 15–24 age-group. Later in this paper, we explore the determinants of the ages at marriage. For now, the key observation is that there has been a steady increase in the age at marriage throughout India, though there is still considerable under-age marriage in the country.

Fig 1, panel (c) presents the age at first birth. We note a steady rise in this age in the last two rounds of the survey, in both age-groups of surveyed women. Given that this variable is censored for a large proportion of our sample (even larger than the age at marriage) however, we believe that these results cannot be easily interpreted and thus we do not examine the variation across states.

Next, we examine the CPR (Fig 1, panel (d)). As mentioned earlier, this particular indicator presents some challenges of quality across surveys, particularly the fourth round. With that in mind, it is still interesting to note that there has been at best only a modest increase in the utilization of contraception till 2005 and a decline in the final round. This recent decline has been the subject to a great deal of debate [46, 48]. We believe that the issues with data quality in the fourth round are significant–we thus note it here, but do not analyze the data any further at the state level.

We turn instead to look at estimates of sterilization–a metric that is less likely to be misreported (compared to the CPR or MCPR) because the question is asked several times and in several different ways in the survey. Moreover, since sterilization is a salient event in a woman’s life, with significantly greater long-term consequences that utilization of temporary forms of contraception, it is less likely to be misreported. We see in Fig 2, panel (e) quite clearly that sterilization remains a very popular form of contraception in India. Among women aged 25+, close to 40% of women are sterilized in all of the survey rounds. Sterilization levels are negligible for under 25 age-group (<10% in all rounds), which suggests that most sterilization occur for women aged older than 25. The popularity of sterilization in India, particularly in the southern states, has been widely discussed [41–43, 49]. What is noteworthy here is the persistence of the pattern over the states over the entire period of our study (Fig 4): Andhra Pradesh leads the country in sterilization rates in both age-groups for the entire study period, with more than 70 percent of women sterilized. Tamil Nadu and Karnataka close behind. In these states, approximately 15 percent of women aged 15–24 are reporting the use of sterilization. Many states show a contrasting pattern for the two age-groups of women. In Kerala, Himachal Pradesh, Rajasthan, Haryana, Madhya Pradesh, West Bengal, Goa, Maharashtra, Gujarat sterilization rates are 40% or more for women aged 15–49, but well-below 5% for the cohort of younger women. Fig 2, panel (e) does highlight a decrease in the levels of sterilization in both age-groups in the paper. This is confirmed in Fig 4: note that sterilization levels are lower across almost all the states.

Finally, we examine female employment. Fig 2 (panel (e)) illustrates a stable pattern of employment across the states of India for the first three survey rounds, but a decline in the participation of women in all three age-groups considered here in the most recent round. India’s low level of female labor force participation, and the further decline in this variable in recent years has been widely reported elsewhere [14–16, 18]. This chart, and the state-level breakdown presented in Fig 5, however, provides some interesting perspectives on the variations by age-group and state of residence. In no Indian state today do we see more than 25% of women in the age-group of 15–24 working for paid employment. Even in the northeastern states, where have consistently shown higher levels of women’s participation, fewer than one quarter of women aged 15–24 are in the labor force. This is a very different scenario than seen in the previous rounds. While these declines may be driven by a wide variety of factors, it is possible that delays in both school completion and marriage, which presumably result in a later migration to a husband’s home, may interfere with women’s labor market opportunities. Young women in the youngest cohorts in 2015 may simply be failing to gain traction in the labor market compared to their counterparts in older age-groups who married and moved to their husband’s homes early enough to start their trajectories in a single location. Given that marriage in India typically involves a woman migrating from her natal home to her husband’s home, marriage leaves employment trajectories quite disrupted [15, 36].

Taken together, the analysis of these variables at the state-level produces some interesting insights into the lives of Indian women. First, women aged 15–24 all across India are delaying marriage and marrying at older ages than their counterparts in older age-groups. Here, there is surprisingly little variation across states. There is no clear evidence however, that women’s reliance on contraception (particularly modern contraception) or employment opportunities have expanded at the same time. Overall, the average Indian women in all age-groups seems to be less likely to use contraception or work in the formal labor force. There are striking variations in these variables across states. Family-planning and paid employment estimates take their highest values in the southern and northeastern states for women of all ages.

A hypothesis that emerges from this analysis is that for young women in India, completion of secondary school and postponement of marriage to 18 or beyond is compressing reproductive spans relative to older women, i.e. they have a shorter gap between marriage and the first birth compared to women aged 25 and above. This may result in greater barriers to participate in the formal labor force. Female labor force participation, may also be linked to structural issues in the Indian economy, such as changes in demand for skill sets and increased burden of unpaid care due to greater longevity. This pattern varies across states. We explore this in the following section through the analysis of micro-data from the last two rounds of the survey.

### Statistical explorations of recent rounds

**Fig 6:**
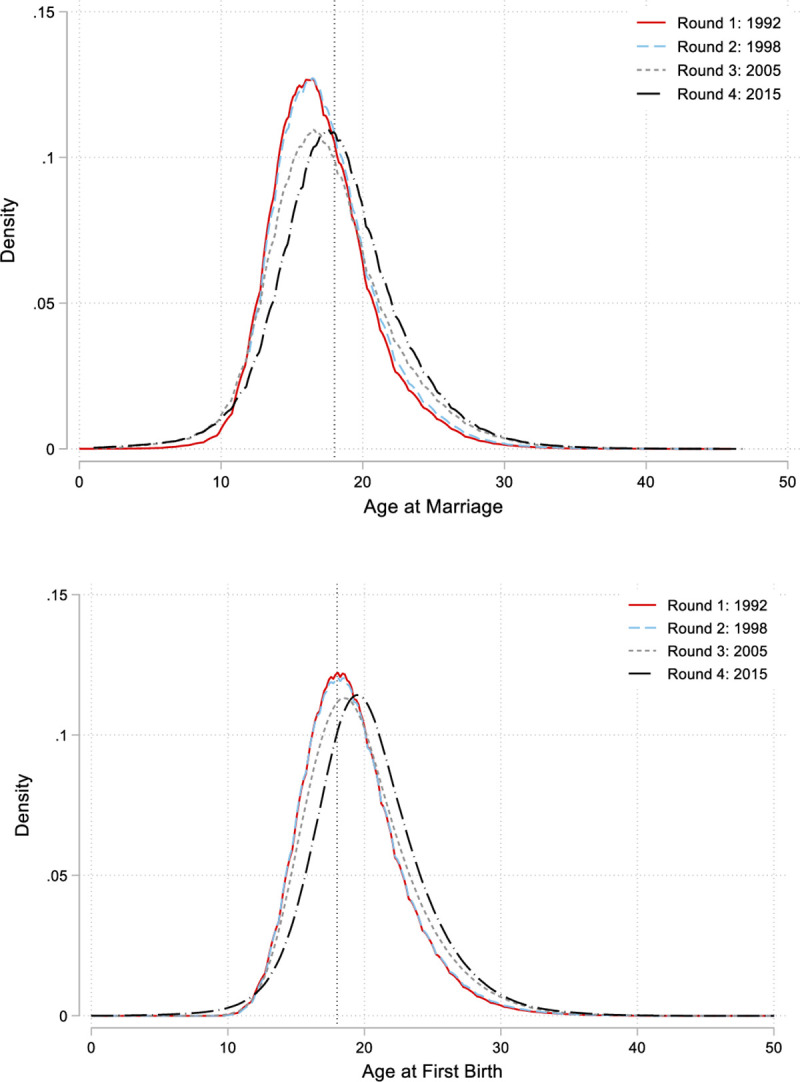
Age at married and age at first birth, conditioned on being married, for all women aged 15–49 in the four survey rounds.

To explore the drivers of the changes of marriage, motherhood and employment at a deeper level, we conduct a micro-analysis of the surveys. Our analysis begins with a deeper look at the distributions of two key variables: the age at marriage and the age at first birth. Kernel density plots for all women, in all four rounds of the survey, are presented in Fig 6. Consistent with the state-level analysis conducted in the previous section, we see that the age at marriage has shifted slightly over the four rounds of the survey. In 1992, the average Indian woman married at the age of approximately 17, while in the NFHS 4, this shifted to just above 18. The age at first birth also increased by 1 year, from 19.1 in 1991 to 20.3 in 2015. The implications of this 1-year increase must be viewed in light of the evidence that delaying marriage and childbirth even by 1 year is associated with increased schooling and has been shown to have a causal impact on women’s investments in their children’s education [35, 36, 50, 51].

We further explore the correlates of women’s key life decisions using a statistical framework. Here we draw on the most recent survey rounds, i.e. NFHS-3 and NFHS-4. The rationale to focus on these surveys is two-fold. First, these two surveys included *all* women, regardless of marital status, in the range of 15–49 (Table 1), while the previous two rounds only focused on married women. Second, as mentioned earlier, the two most recent rounds cover women in the age-group that is critical to India’s current demographic dividend. Given the data issues described earlier, we refrain from pooling the data and run separate regressions for the two survey rounds.

We use a simple linear regression model, with a set of interaction terms, to understand the determinants of demographic outcomes for:

$$\begin{array}{l} y_{ig}=\sum_{g=1}^{2}\beta_{1g}\;\mathrm{Age}\;18\mathrm{to}24\times \mathrm{Minority}\;{\mathrm{Group}}_{g}\times \mathrm{NoSec}Sch_{i}+\sum_{g=1}^{2}\beta_{2g}\;\mathrm{Age}\;18\mathrm{to}24\times \mathrm{Minority}\;{\mathrm{Group}}_{ig}\\ \;+\sum_{g=1}^{2}\beta_{3g}\;\mathrm{Minority}\;{\mathrm{Group}}_{g}\times {\mathrm{NoSecSch}}_{i}+\sum_{g=1}^{2}\beta_{4g}\;\mathrm{Minority}\;{\mathrm{Group}}_{ig}\\ \;+\beta_{5}\left(Age18to24_{i}\times No\mathrm{Sec}Sch_{i}\right)+\beta_{6}\;\mathrm{NoSec}Sch_{i}+\beta_{7}\;Age18to24_{i}+\gamma X_{ig}+\delta_{s}+\varepsilon_{ig} \end{array}$$

where *y*_*isr*_ is the outcome variable for individual *i*, residing in state *s* and group *g*, where *g* is a dummy variable that identifies Muslims or members of “scheduled castes” or “scheduled tribes” (this is abbreviated as *SCST)*. The Scheduled Caste (SCs) and Scheduled Tribes (STs) are groups of people in India who are recognized as disadvantaged by the constitution of India. We consider several dependent variables: marriage, marriage below the age of 18, and the length, in months, of the first birth interval, i.e. the time between marriage and first birth (for those who are married). In keeping with the conceptual framework and the results presented earlier, we also examine a woman’s employment status, reliance on modern contraception and sterilization status. Robust standard errors are calculated at the psu–this is largely because district identifiers are unavailable in the 2005, Round 3 data.

Earlier we presented summary statistics for the group aged 15–24 (Ages15to24) to contrast the youngest cohorts of India’s women with those older than 25. In light of the findings regarding age at marriage however, we restrict statistical analysis for the youngest age-group to 18–24. This is for three reasons. First, 18 is the legal minimum age at marriage in India. Understanding the determinants of achieving this threshold is of interest in its own right. Second, given that age-misreporting remains a concern in NFHS data, we believe that the use of the legal threshold makes our results less sensitive to the distribution of reported ages [52]. Third, the most recent survey suggests that the average age at marriage for younger women has now crossed 18, so observations below that threshold are “censored” i.e. women below this age *may* marry before 18 but have not yet married.

Our model also features several control variables. *X*_*ist*_ is a vector of controls that include simple measures of education, religion, caste and social status. Education is measured simply by a dummy variable that takes value 1 if a woman does not complete secondary school, and 0 otherwise (this variable is abbreviated to *NoSecSch*). We interact age-dummies with the education measure as well as the SCST measure to better understand the role of caste and education in the youngest population of women. We also include separate dummy variables for being Muslim. *δ*_*s*_ is the state-level fixed effect. We perform separate regressions for different regions of India, to better understand the regional variations in the lives of women.

**Fig 7:**
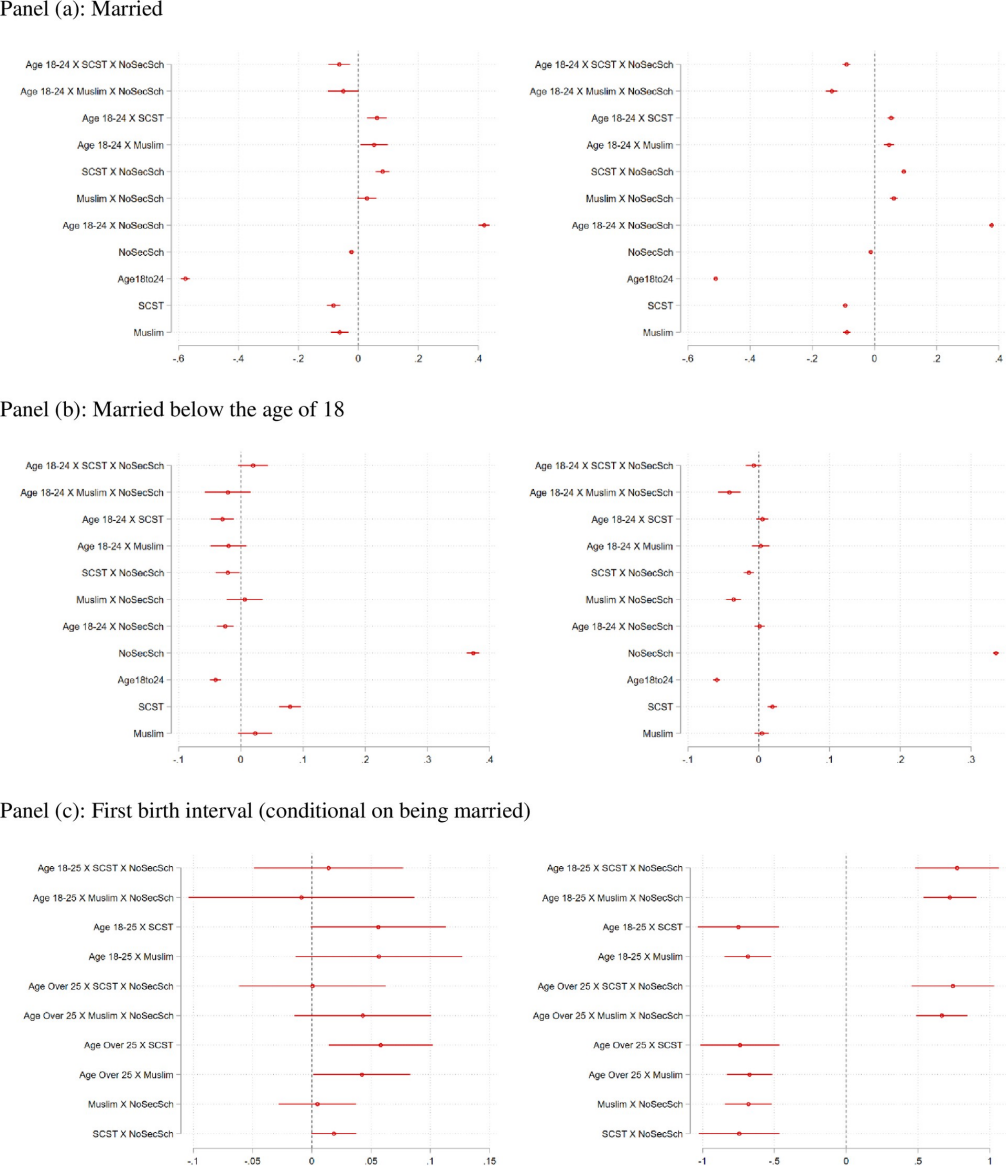
Coefficients, with confidence intervals, for regressions of key outcomes: The dummy variable for marriage below the age of 18 (dependent variable, measured in months), age at first birth, and the first birth interval on age intervals as well as a set of control variables that include schooling (as measured by completion of secondary school), dummies for religious groups, dummies for caste groups and state-level fixed-effects. The left panel depicts estimates for the regressions with the sample from Round 3 (2005–06) and the right panel shows the estimates from round 4 (2015–16). The excluded group in all regressions is the group above than the age of 40.

Results are presented in graphical form in Fig 7. Panels (a) and (b) of Fig 7 presents the regression coefficients of the regression with the dependent variable “Married” and “Married before the age of 18”. We present the regression coefficients in visual form and emphasize that the omitted group in these regressions is the age-group above the age of 25. The left panel presents results from the NFHS 3, and the right panel presents results from the NFHS 4. Full results from the regressions, including the full effects of the specific variables featured in the interaction terms, as well as F-tests for the joint significance of the “groups” of variables–age, schooling, SCST and Muslim–are presented in Tables 3 and 4 for Rounds 3 and 4 respectively. Again, we note that we do not pool these datasets because of the differences in survey design and the concerns about consistency of data-quality that pertain to many of our variables, particularly contraception and employment.

**Table 3:** Determinants of regression results from NFHS-3.

	Ever Married	Married Before 18	First birth Interval	Use any contraception	Sterilized	Employed
	(1)	(2)	(3)	(4)	(5)	(6)
**Age 18–24 × SCST × NoSecSch (*β***_**1,*SCST***_**)**	-0.064*	0.019	-0.030	-0.022	0.069***	0.085***
	(0.025)	(0.018)	(1.308)	(0.015)	(0.011)	(0.021)
**Age 18–24 × Muslim × NoSecSch (*β***_**1,*Muslim***_**)**	-0.051	-0.021	-0.772	0.052*	0.131***	0.013
	(0.048)	(0.030)	(1.241)	(0.022)	(0.017)	(0.027)
**Age 18–24 × SCST (*β***_**2,*SCST***_)	0.062**	-0.030*	0.514	0.106***	-0.003	-0.141***
	(0.017)	(0.012)	(1.155)	(0.014)	(0.010)	(0.020)
**Age 18–24 × Muslim** (***β***_**3,*Muslim***_)	0.052	-0.020	2.117	0.119***	0.048**	0.039
	(0.036)	(0.021)	(1.124)	(0.020)	(0.015)	(0.025)
**SCST × NoSecSch** (***β***_**3,*SCST***_)	0.081***	-0.021	-0.865	0.000	-0.089***	-0.053***
	(0.014)	(0.036)	(0.825)	(0.013)	(0.010)	(0.016)
**Muslim × NoSecSch** (***β***_**3,*Muslim***_)	0.028	0.006	-0.531	-0.057**	-0.125***	-0.019
	(0.032)	(0.030)	(1.212)	(0.020)	(0.016)	(0.020)
**SCST** (***β***_**4,*SCST***_)	-0.083***	0.079**	0.131	-0.093***	0.037***	0.158***
	(0.012)	(0.028)	(0.681)	(0.012)	(0.009)	(0.015)
**Muslim** (***β***_**4,*Muslim***_)	-0.063*	0.023	-2.239**	-0.143***	-0.085***	-0.091***
	(0.025)	(0.025)	(0.711)	(0.020)	(0.016)	(0.020)
**Age 18–24 × NoSecSch (*β***_**5**_**)**	0.420***	-0.025	-0.736	0.173***	-0.101***	0.062***
	(0.027)	(0.013)	(0.681)	(0.008)	(0.006)	(0.009)
**NoSecSch (*β***_**6**_**)**	-0.023*	0.374***	4.611***	-0.117***	0.140***	-0.018*
	(0.011)	(0.029)	(0.782)	(0.006)	(0.005)	(0.007)
**Age18to24 (*β***_**7**_**)**	-0.578***	-0.041***	-5.112***	-0.354***	-0.103***	-0.093***
	(0.011)	(0.008)	(0.540)	(0.007)	(0.005)	(0.008)
**Parity**				0.097***	0.075***	0.021***
				(0.001)	(0.001)	(0.001)
**Constant**	0.417***	0.074***	0.321***	0.417***	0.074***	0.321***
	(0.006)	(0.005)	(0.006)	(0.006)	(0.005)	(0.006)
**R-Squared**	0.264	0.283	0.067	0.264	0.283	0.067
**N**	124385	124385	124385	124385	124385	124385
**Estimate: Age-18-24**	-0.098***	0.143***	-0.097***	-0.098***	0.143***	-0.097***
**se**	(0.015)	(0.012)	(0.016)	(0.015)	(0.012)	(0.016)
**Estimate: NoSecSch**	0.029*	0.025**	0.069***	0.029*	0.025**	0.069***
**se**	(0.016)	(0.012)	(0.025)	(0.016)	(0.012)	(0.025)
**Estimate: SCST**	-0.008	0.014***	0.049***	-0.008	0.014***	0.049***
**se**	(0.006)	(0.004)	(0.008)	(0.006)	(0.004)	(0.008)
**Estimate: Muslim**	-0.029***	-0.031***	-0.058***	-0.029***	-0.031***	-0.058***
**se**	(0.008)	(0.006)	(0.012)	(0.008)	(0.006)	(0.012)
**F test of joint significance:Age 18–24**	659.185	178.171	80.360	659.185	178.171	80.360
**p-value**	0.000	0.000	0.000	0.000	0.000	0.000
**F test of joint significance:NoSecSch**	124.196	134.673	18.468	124.196	134.673	18.468
**p-value**	0.000	0.000	0.000	0.000	0.000	0.000
**F test of joint significance:SCST**	72.695	44.288	55.035	72.695	44.288	55.035
**p-value**	0.000	0.000	0.000	0.000	0.000	0.000
**F test of joint significance:Muslim**	118.753	158.216	16.467	118.753	158.216	16.467
**p-value**	0.000	0.000	0.000	0.000	0.000	0.000

**Table 4:** Determinants of regression results from NFHS-4.

	Ever Married	Married Before 18	First birth Interval	Use any contraception	Sterilized	Employed
	(1)	(2)	(3)	(4)	(5)	(6)
**Age 18–24 × SCST × NoSecSch (*β***_**1,*SCST***_)	-0.090***	-0.007	0.011	-0.030***	0.026***	0.050***
	(0.007)	(0.006)	(0.448)	(0.005)	(0.004)	(0.014)
**Age 18–24 × Muslim × NoSecSch (*β***_**1,*Muslim***_)	-0.138***	-0.042***	-0.216	0.034***	0.111***	0.061***
	(0.010)	(0.008)	(0.568)	(0.008)	(0.005)	(0.018)
**Age 18–24 × SCST (*β***_**2,*SCST***_)	0.053***	0.005	0.736	0.079***	0.026***	-0.073***
	(0.006)	(0.004)	(0.396)	(0.005)	(0.003)	(0.013)
**Age 18–24 × Muslim (*β***_**2,*Muslim***_)	0.046***	0.003	1.177*	0.095***	0.094***	0.046**
	(0.008)	(0.006)	(0.494)	(0.007)	(0.005)	(0.016)
**SCST × NoSecSch (*β***_**3,*SCST***_)	0.094***	-0.014***	-0.717**	0.002	-0.048***	-0.028**
	(0.004)	(0.004)	(0.251)	(0.004)	(0.003)	(0.010)
**Muslim × NoSecSch (*β***_**3,*Muslim***_)	0.062***	-0.036***	-0.388	-0.052***	-0.128***	-0.062***
	(0.006)	(0.005)	(0.339)	(0.006)	(0.005)	(0.013)
**SCST (*β***_**4,*SCST***_)	-0.095***	0.019***	0.823***	-0.073***	0.002	0.098***
	(0.004)	(0.003)	(0.229)	(0.004)	(0.003)	(0.010)
**Muslim (*β***_**4,*Muslim***_)	-0.089***	0.004	-1.808***	-0.113***	-0.081***	-0.022
	(0.006)	(0.005)	(0.315)	(0.006)	(0.004)	(0.013)
**Age 18–24 × NoSecSch (*β***_**5**_)	0.376***	0.001	-1.879***	0.097***	-0.096***	0.043***
	(0.004)	(0.004)	(0.241)	(0.003)	(0.003)	(0.008)
**NoSecSch (*β***_**6**_)	-0.012***	0.335***	5.541***	-0.031***	0.121***	-0.030***
	(0.002)	(0.002)	(0.130)	(0.003)	(0.002)	(0.005)
**Age18to24 (*β***_**7**_)	-0.511***	-0.059***	-6.408***	-0.247***	-0.095***	-0.091***
	(0.003)	(0.002)	(0.197)	(0.003)	(0.002)	(0.006)
**Parity**				0.101***	0.076***	0.021***
				(0.001)	(0.001)	(0.001)
**Constant**	0.847***	0.177***	24.903***	0.291***	0.066***	0.219***
	(0.002)	(0.002)	(0.112)	(0.003)	(0.002)	(0.005)
**R-Squared**	0.134	0.125	0.052	0.246	0.258	0.051
**N**	699686	697350	437750	699686	699686	122351
**Estimate: Age-18-24**	-0.641***	-0.101***	-4.698***	-0.069***	0.161***	-0.007
**se**	(0.007)	(0.008)	(0.445)	(0.006)	(0.005)	(0.014)
**Estimate: NoSecSch**	0.291***	0.237***	2.354***	0.021***	-0.013***	0.034**
**se**	(0.009)	(0.007)	(0.564)	(0.005)	(0.003)	(0.015)
**Estimate: SCST**	-0.038***	0.003	0.854***	-0.022***	0.006***	0.047***
**se**	(0.003)	(0.004)	(0.186)	(0.002)	(0.002)	(0.007)
**Estimate: Muslim**	-0.119***	-0.070***	-1.234***	-0.036***	-0.004*	0.023***
**se**	(0.005)	(0.005)	(0.262)	(0.004)	(0.002)	(0.009)
**F test of joint significance:Age 18–24**	7603.168	186.458	281.420	2043.295	1215.166	119.178
**p-value**	0.000	0.000	0.000	0.000	0.000	0.000
**F test of joint significance: NoSecSch**	3317.097	8130.043	460.614	263.458	637.866	25.065
**p-value**	0.000	0.000	0.000	0.000	0.000	0.000
**F test of joint significance:SCST**	259.598	26.412	15.495	204.298	193.707	38.951
**p-value**	0.000	0.000	0.000	0.000	0.000	0.000
**F test of joint significance:Muslim**	164.480	19.941	14.771	423.859	1492.757	54.526
**p-value**	0.000	0.000	0.000	0.000	0.000	0.000

Several interesting results emerge from the analysis. Panels (a) and (b) present the regression coefficients for the two dependent variables that pertain to marriage (*EverMarried* and *MarriedBef18*). We discuss the coefficients in order of their magnitude and significance. Note that *β*_5_–the coefficient for the interaction term *Age*18−24 × *NoSecSch—*is positive and significant for the “Ever Married” regression in both rounds, and *β*_6_—the coefficient for *NoSecSch–*is positive and significant in the “Married before the age of 18” regression. Precise coefficients are seen in Tables 3 and 4. These positive coefficients suggest that the lack of completion of secondary schooling is one of the biggest predictors of early marriage for young women today. As seen in Table 3, the overall total effect of the schooling variable, calculated by adding up the interaction terms that feature this variable, suggest that women who do not complete secondary schooling are 39 and 29 percentage points more likely to be married before the age of 18 in Rounds 3 and 4 (compared to women aged 25 and older). The results are statistically significant at the 1% level. The effect of incomplete schooling declined between 2005 and 2015, but this is a striking result even in the later round.

It is also noteworthy that *β*_1,*SCST*_ and *β*_1,*Mulsim*_, the coefficients for the triple interaction terms represented as *Age*18*to*24×*SCST*×*NoSch* and *Age*18*to*24×*Muslim*×*NoSch* respectively, are *negative* and statistically significant in both rounds of the survey, suggesting that even the most disadvantaged women, are *not* more likely to be married, or married before the age of 18 than the average woman aged 25 and above. The double interaction terms in the first graphs (left hand side) of panels (a) and (b) however, suggest that *β*_2,*SCST*_ and *β*_2,*Mulsim*_, i.e. the interaction terms *Age*18*to*24×*SCST* and *Age*18*to*24×*Mulsim* are *positive* and statistically significant, suggesting that young women from these groups are more likely to be married than the average Indian woman older than 25. Estimates presented in Table 3 suggest that in Round 3, when compared to non-SCST and older women, SCST women aged 18–24 are about 6 percentage points more likely to be married but 3 percentage points less likely to be married before the age of 18. In Round 4, SCST women aged 18–24 are about 5 percentage points more likely (than the comparison group) to be married there is not statistically significant effect in the regression for marriage before the age of 18. These estimates are not significant for Muslim women in Round 3, and the coefficients in Round 4 are smaller in magnitude than observed for SCST women. The two results together suggest that that SCST women are likely to marry close to the threshold of 18. To summarize, the important insight here is that the biggest determinant of early marriage is the lack of completion of secondary school. While SCST women also marry early relative to their peers, the SCST effect is small in comparison to the effect of educational disadvantage.

Panel (c) of Fig 7 presents the regression coefficients of the regression with the dependent variable “First birth interval (in months)”. As in the case above, results for survey rounds 3 and 4 are on the left and right respectively. Again, we present the interaction effects of age with caste, and also age with lack of completion of secondary school. Few variables emerge as significant here but again we see that the lack of completion of secondary school, and SCST status, is associated with longer birth intervals. The combined effect of the age dummy *Age18-24* is -3.283 in Round 3 and -4.698 in Round 4 (both are significant at the 1% level) which suggests that those women who *do* marry in this group are actually shortening the transition to motherhood. We emphasize however, that our birth interval is measured in months–this indicates that early marriages in this age-group do not result in significant increases in the months to motherhood. We also concede that these results are difficult to interpret because they are conditional on marriage and thus conducted on a smaller sample of women in both rounds of the survey.

**Fig 8:**
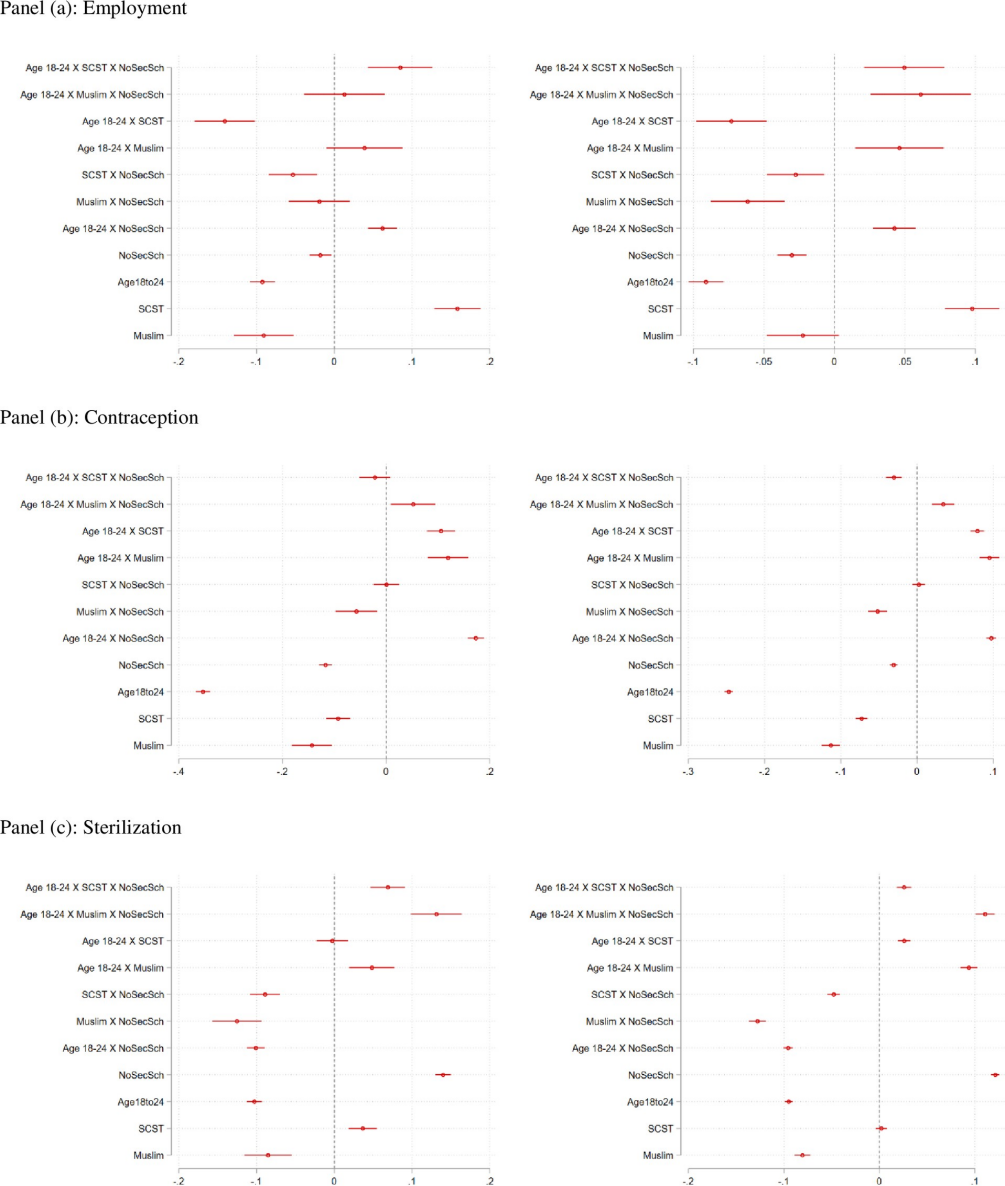
Coefficients, with confidence intervals, for regressions of the prevalence of female sterilization (as measured by a dummy variable) on age intervals as well as a set of control variables that include age at marriage, schooling (as measured by completion of secondary school), dummies for religious groups, dummies for caste groups and state-level fixed-effects. The excluded group in all regressions is the group above than the age of 40.

Panel (a) of Fig 8 presents the results for employment. In both rounds of the survey we see that the coefficient for the variable SCST (*β*_6,*SCST*_) is positive and significant. The magnitudes of the effects, seen more clearly in the combined effects listed at the bottom of Tables 3 and 4, are quite noteworthy: compared to non-SCST women, SCST women are 10 percentage points more likely to be employed in Rounds 3 and 7 percent more likely to be employed in Round 4. These effects are bigger than the effects of schooling disadvantage. Compared to those with secondary schooling, women who fail to complete secondary school are 7 percentage points more likely to work in Round 3 and 3.4 percentage points more likely to work in Round 4. Interestingly *β*_1,*SCST*_ and *β*_1,*Muslim*_, the coefficients for the triple interaction terms represented as *Age*18*to*24×*SCST*×*NoSch* and *Age*18*to*24×*Muslim*×*NoSch* respectively, feature coefficients that are much smaller in magnitude and not statistically significant (with the exception of *β*_1,*SCST*_ in Round 4, which has a magnitude of 5 percentage points and is statistically significant at the 10% level).

These results suggest that even in the cohort of the younger women aged 18–24, SCST women are more likely to marry young and also more likely to work than their higher-status counterparts over the age of 25. This finding is quite striking in light of the fact presented earlier–the declining overall level of female employment in India. For SCST women, particularly those without an education, higher labor force participation rate may be the result of economic hardship rather than an empowered choice. More work is needed to understand this result, but it is consistent with other recent studies that highlight the vulnerability of this group, particularly in rural areas [15, 16, 39].

Panels (b) and (c) of Fig 8 present the regression results for the usage of any form of contraception and the practice of sterilization respectively. The biggest insight from these two sets of regression results is in the contrast between younger cohorts of women in disadvantaged groups and the rest of their cohorts, even when all are compared against the women over the age of 25. *β*_2,*SCST*_ and *β*_2*Muslim*_–coefficients for the interaction terms *Age*18*to*24×*SCST* and *Age*18*to*24×*Muslim* are–are positive and statistically significant in both rounds of the regressions. Overall however, women aged *Age*18*to*24 are 9.8 and 6.7 percentage points less likely to use contraception in Round 3 and 4 respectively. This may likely be driven by the large percentage of women in this age-group who are not yet married. Alternately, it may be the result of higher levels of labor force participation in these groups. In results not shown here, we restrict the sample to those women who are married and find a *higher* level of utilization of contraception for this age-group conditional on being married.

In the regression for sterilization, we note that *β*_1,*SCST*_ and *β*_1,*Muslim*_ are positive and statistically significant at the 1% level in both rounds of the survey. This suggests that when compared to the comparison group (women older than 25, not in the specified minority group and have some secondary schooling), SCST and Muslim women aged 15–24 are 7 and 13 percentage points more likely to be sterilized in Round 3 (Table 3). *β*_1,*Muslim*_ continues to be positive and significant in Round 4 (Table 4) with a magnitude of 0.11. The positive coefficients however, are just concentrated in the group with educational disadvantage. The full-effects of being Muslim, presented at the bottom of the two tables, show that Muslims are *overall less* likely than non-Muslims, to be sterilized in the two rounds of the survey. The lower incidence of sterilization among Muslims declines in magnitude by the most current round to an estimate that is close to zero, but it nevertheless remains a statistically significant estimate. This is a surprising finding, particularly considering that many women in this age-group are not yet married.

All the results in our statistical model are obtained from models with psu-level fixed effects. This suggests that regardless of their location, the life trajectories of Indian women today remain deeply affected by their levels of education, caste and religion. The completion of secondary school affects the timing of marriage. Caste and religion, however, affect subsequent decisions such as the timing of birth, employment, and usage of contraception. For some outcomes such as employment, educational disadvantages *exacerbate* social disadvantages.

In results not shown here, we run these regressions separately for different regions of India, with fixed-effects for states. We omit these results to conserve space, but simply highlight that the regional variations have declined dramatically over the two rounds of the survey and in almost all the regions, the key results for young women (18–24) relative to older women remain the same: the age at marriage is climbing but early marriage remains a common practice in some areas (particularly the states of Bihar, Rajasthan, Uttar Pradesh, Madhya Pradesh and Jharkhand), early marriage is highly correlated with the lack of completion of schooling and not driven by minority status in disadvantaged groups, the birth interval between marriage and motherhood is however, longer in early marriages among the youngest women, employment is highly concentrated in SCST women and the practice of contraception has been favored by the youngest age-groups.

## Summary and conclusion

This paper uses four major national population surveys that span the years 1998–2016 to construct a broad descriptive overview of the current status and recent changes in the demographic and economic indicators pertinent to India’s young women. Data from the third and fourth wave of the National Family Health Survey, collected in 2005–06 and then 2015–16 are analyzed in depth.

We have analyzed the data at the macro- as well as the micro-level. Taken together, the results suggest that India’s youngest women continue to marry and have their first births quite early: the ages at marriage and first birth are approximately 18.2 and 20.3 respectively, and these have increased by only about 18 months in the past 20 years. Though only 20% of Indian women aged 15–50 have completed secondary school, this estimate is 34% for women aged 18–24. Women who drop out of secondary school are more likely to marry before the age of 18, give birth before the age of 18, and receive sterilization surgeries once they are done with childbearing. Our descriptive analysis shows however, that there are sharp variations in these variables across states. Southern states such as Kerala, Tamil Nadu, Andhra Pradesh and Karnataka showed higher ages at marriage, education completion rates, and contraceptive throughout the surveys, though other regions such as the Northeast do appear to demonstrate some gains, particularly between Rounds 3 and 4.

Another noteworthy result from our analysis is that while education seems to result in delays in marriage, education has weaker effects on the timing of births, employment patterns or the utilization of contraception. The age at first birth has risen very slowly and statistical models show little effects of education, caste or religion on the first birth interval, i.e. the number of months between marriage and birth of a first child. SCST women however, do seem to have increased birth intervals, but the effect is quite modest.

While the lives of Indian women are definitely affected by where they are born, our results also indicate that micro-variables such as education, caste and religion, which vary considerably within states and communities, continue to play a role in the life trajectories of Indian women, including the youngest age-groups. While education has the biggest influence on the *timing* of marriage, caste and religion loom large into other decisions. Educational disadvantages often combine with these caste identities. Women from disadvantaged communities are the most likely to participate in the labor force, even in the youngest cohort. The lack of completion of secondary school exacerbates this effect, i.e. disadvantaged women do not complete secondary school are even likely to report that they participate in the labor force. This is particularly true in the youngest cohort aged 18–24 years. Caste and religion also affect the usage of contraception. Our descriptive analysis suggests that the usage of modern contraception by women in the age-group 15–24 has also been declining but the 18–24 cohort does report a statistically higher utilization rate than their counterparts above the age of 25.

Taken together, these results show that young women’s life trajectories in India today depend quite a bit on *where* they live as well as on their caste and religion. Disadvantaged women tend to delay marriage and motherhood, drop out of secondary school, and participate in the labor force. For higher status groups, paradoxically, the first birth interval is compressed, labor force participation is lower, and utilization of contraception also lower than their older peers. This suggests that for women with high status, marriage and motherhood are more salient events, there is pressure to bear children quickly after marriage and less emphasis on labor market opportunities.

These results have important implications for India’s demographic transition and demographic dividend. It is clear that investments in women’s opportunities in the form of education, access to contraception and employment have been uneven across India. Though the effects of caste, religion and tribal status have diminished in recent years, patterns of disadvantage remain stubborn in many parts of India. Too many women remain caught in a cycle of dropping out of secondary school, marrying early, failing to access modern contraception, giving birth early, and never having an opportunity to enter the formal labor force. Regional differences persist but caste and religion matter everywhere.
